# *De Novo* Transcriptome Sequencing of Low Temperature-Treated *Phlox subulata* and Analysis of the Genes Involved in Cold Stress

**DOI:** 10.3390/ijms16059732

**Published:** 2015-04-29

**Authors:** Yanting Qu, Aimin Zhou, Xing Zhang, Huanwei Tang, Ming Liang, Hui Han, Yuhu Zuo

**Affiliations:** 1 College of Agriculture, Heilongjiang Bayi Agricultural University, Daqing 163000, China; E-Mail: quyanting1976@126.com; 2 Institute of Natural Resources and Ecology, Heilongjiang Academy of Sciences (HAS), Collaborative Innovation Center for Development and Utilization of Forest Resources, Harbin 150040, China; E-Mails: zhangxing0821@vip.sina.com.cn (X.Z.); tanghw1968@126.com (H.T.); liangming2013_8@163.com (M.L.); ziyuan-han@163.com (H.H.); 3 College of Horticulture, Northeast Agricultural University, Harbin 150030, China; E-Mail: zhouaimin@aliyun.com

**Keywords:** *Phlox subulata*, cold acclimation, transcriptome, differentially expressed genes

## Abstract

*Phlox subulata*, a perennial herbaceous flower, can survive during the winter of northeast China, where the temperature can drop to −30 °C, suggesting that *P. subulata* is an ideal model for studying the molecular mechanisms of cold acclimation in plants. However, little is known about the gene expression profile of *P. subulata* under cold stress. Here, we examined changes in cold stress-related genes in *P. subulata*. We sequenced three cold-treated (CT) and control (CK) samples of *P. subulata*. After *de* *novo* assembly and quantitative assessment of the obtained reads, 99,174 unigenes were generated. Based on similarity searches with known proteins in public protein databases, 59,994 unigenes were functionally annotated. Among all differentially expressed genes (DEGs), 8302, 10,638 and 11,021 up-regulated genes and 9898, 17,876, and 12,358 down-regulated genes were identified after treatment at 4, 0, and −10 °C, respectively. Furthermore, 3417 up-regulated unigenes were expressed only in CT samples. Twenty major cold-related genes, including transcription factors, antioxidant enzymes, osmoregulation proteins, and Ca^2+^ and ABA signaling components, were identified, and their expression levels were estimated. Overall, this is the first transcriptome sequencing of this plant species under cold stress. Studies of DEGs involved in cold-related metabolic pathways may facilitate the discovery of cold-resistance genes.

## 1. Introduction

Low temperature and cold are major environmental stressors that can influence the distribution, growth, and development of plants. Cold acclimation is the main process involved in increasing cold and freezing tolerance of some temperate plant species under chilling and freezing conditions. During this process, extensive physiological and biochemical changes occur in plants, allowing them to withstand low temperature conditions [1]. These changes are mediated through the differential expression of many genes responding to cold stress [2]. Detection and identification of these genes are important for understanding the regulatory mechanisms of cold acclimation [3]. For example, in the model plant *Arabidopsis thaliana*, many transcription factors that are involved in the regulation of cold-related genes have been identified; the most well-studied pathways are regulated by the transcription factors DREB/CBF (dehydration-responsive-element-binding/C-repeat binding factors), which promote the expression of downstream genes by specific binding to the DRE/CRT (dehydration-responsive-element/C-repeat) *cis*-elements in the promoter regions of target genes [4]. Recent studies have also identified upstream regulators of DREB/CBF, including ICE1, MYB15, CAMTA and HOS1 [5,6,7,8].

Unlike model plants, many non-model plant species are able to grow in specific environments because of their specialized genetic resources and adaptive mechanisms to native adverse conditions. Moreover, with the advent of next-generation sequencing technology (e.g., Illumina/Solexa-based RNA-seq) [9], large-scale transcriptome data have become available in non-model species. Furthermore, such technologies allow for high accuracy and sensitivity, even for genes with low expression levels, and permit rapid identification and analysis of whole transcriptomes. Currently, Illumina/Solexa has been successfully applied for transcriptome sequencing in many plant species, including *Cunninghamia lanceolata* [10], *Raphanus sativus* [11], *Ammopiptanthus mongolicus* [12], *Anthurium andraeanum* [13] and *Wolfiporia cocos* [14].

*Phlox subulata* L. is a perennial herbaceous flower and a member of the Polemoniaceae family. This plant has strong resistance to salt, drought, heat, and cold stresses. *P. subulata* can survive safely during the winter at temperatures as low as −40 °C, maintaining a green phenotype at −12 °C. The ability to survive to such low temperatures suggests that *P. subulata* may have distinctive molecular mechanisms that adapt to cold stress conditions. However, to date, no previous genomic information has been reported in *P. subulata* plants.

In this study, we aimed to examine the genomic characteristics of *P. subulata* that confer resistance to cold. To this end, we sequenced and annotated the transcriptome of *P. subulata* under normal and cold treatment conditions using RNA-seq and publicly available databases. We also analyzed differentially expressed genes (DEGs) of cold-treated (CT; 4, 0 and −10 °C) and control (CK) plants, and identified numerous specific cold-related genes. Our results provided a foundation for understanding the cold response mechanism of *P. subulata* and provided a valuable resource for the development of cold-tolerant plants through genetic manipulation.

## 2. Results and Discussion

### 2.1. Phlox subulata (P. subulata) Transcriptome Sequencing and de Novo Assembly

**Figure 1:**
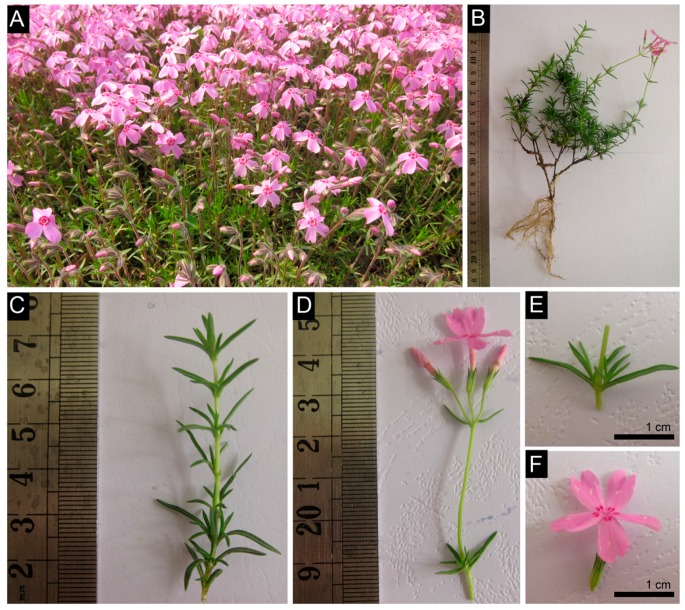
Phenotype characteristics of *Phlox subulata.* (**A**) Natural populations of *P*. *subulata* plants distribution in northeast China; (**B**−**F**) Phenotypes of the *P*. *subulata* plant, and its roots, stems (**B**); leaves (**C**,**E**); and flowers (**D**,**F**); Scale bars = 1 cm (**E**,**F**).

*P. subulata* plants grew and blossomed in the spring and autumn (Figure 1A), producing a 10–15 cm high plant with old stem half-lignification (Figure 1B), needle-like and leathery leaves, and 2 cm pink flowers (Figure 1C–F). Sequence analysis and assembly were performed to investigate the transcriptome and gene expression profiles of *P. subulata* under normal and cold stress. Four cDNA samples from seedlings of CT (4, 0 and −10 °C, subsequently referred to as CT1, CT2 and CT3, respectively) and CK (20 °C) plants were sequenced using an Illumina HiSeq 2000 platform. In total, we obtained approximately 55–59 million raw reads for CT and CK samples. After removing the low-quality reads and reads containing adaptors, 21.3 × 10^7^ clean reads consisting of 19.2 × 10^9^ nucleotides (nt) were obtained with a Q20 percentage (an error probability of 0.01) of more than 97% for four samples (Table 1). All clean reads were deposited in the NCBI Sequence Read Archive (SRA, http://www.ncbi.nlm.nih.gov/Traces/sra) database with accession number SRP055942.

**Table 1:** Statistics of the sequencing and assembly of cold-treated (CT) and control (CK) *Phlox subulata* plants.

Samples	CK (20 °C)	CT1 (4 °C)	CT2 (0 °C)	CT3 (−10 °C)	Total
Total Raw Reads	59,580,728	59,263,032	55,328,170	55,583,970	–
Total Clean Reads	55,528,592	55,319,076	51,409,114	51,736,252	–
Total Clean Nucleotides (nt)	4,997,573,280	4,978,716,840	4,626,820,260	4,656,262,680	–
Q20 percentage	97.50%	97.53%	97.41%	97.51%	–
N percentage	0.01%	0.01%	0.01%	0.01%	–
GC percentage	46.40%	46.32%	46.85%	46.95%	–
**Contig**					
Total Number	126,166	125,583	120,123	140,329	–
Total Length (nt)	48,095,639	47,695,976	42,622,577	48,874,606	–
Mean Length (nt)	381	380	355	348	–
N50	820	857	714	705	–
**Unigene**					
Total Number	82,948	81,059	75,181	85,491	99,174
Total Length (nt)	67,146,823	63,741,994	54,402,959	60,415,665	98,892,318
Mean Length (nt)	810	786	724	707	997
N50	1470	1434	1369	1322	1622
Total Consensus Sequences	82,948	81,059	75,181	85,491	99,174
Distinct Clusters	30,546	29,615	23,876	27,448	42,007
Distinct Singletons	52,402	51,444	51,305	58,043	57,167

**Figure 2:**
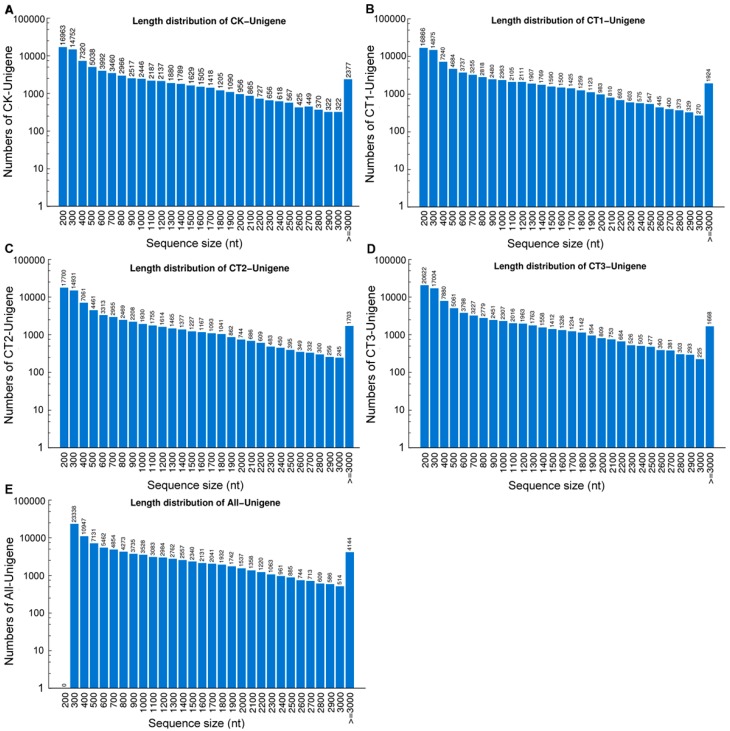
Length distributions of the unigenes from cold-treated (CT) and control (CK) samples. (**A**−**E**) The length distributions of unigenes from CK (**A**); CT1 (**B**); CT2 (**C**); CT3 (**D**); and all samples (**E**).

Transcriptome *de novo* assembly was performed using Trinity program [15]. All high-quality clean reads of each sample were assembled into 125,583 (CT1), 120,123 (CT2), 140,329 (CT3) and 126,166 (CK) contigs, respectively (Table 1). In four samples, the average contig length exceeded 340 nt (length distributions of these contigs are shown in Figure S1). The contigs of each sample were then joined into unigenes, generating 81,059 (CT1), 75,181 (CT2), 85,491 (CT3) and 82,948 (CK) unigenes, respectively. After long-sequence clustering of four samples, a total of 99,174 unigenes were obtained for all samples. The total length was 98,892,318 nt, with a mean length of 997 nt and an N50 of 1622 nt (Table 1). The length distributions of unigenes of each sample are given in Figure 2.

### 2.2. Functional Annotation and Classification of the Assembled Unigenes

**Figure 3:**
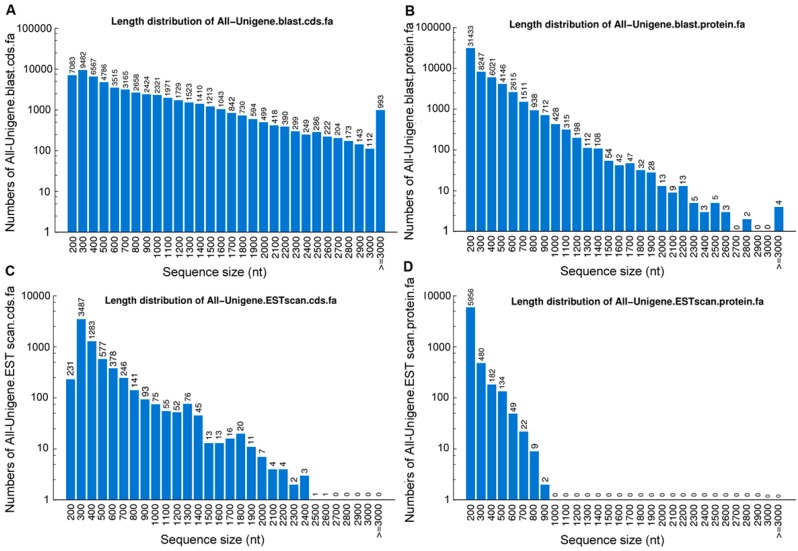
Length distributions of the protein-coding region predictions (CDSs) (**A**−**D**). The length distributions of CDSs using BLASTx (**A**); the length distributions of proteins using BLASTx (**B**); the length distributions of CDSs using ESTscan (**C**); the length distributions of proteins using ESTscan (**D**).

To validate and annotate the assembled unigenes, sequence similarity searches were conducted using sequence- and domain-based alignments. In total, 99,174 unigenes from all groups significantly matched a sequence in at least one of the public databases, including NCBI non-redundant protein (NR), Swiss-Prot protein, Kyoto Encyclopedia of Genes and Genomes (KEGG), Clusters of Orthologous Groups (COG), and Gene Ontology (GO) databases. There were 59,994 unigenes (60.49% of all unigenes) with homologous sequences in at least one of the aforementioned databases (Table 2). Among them, 55,996 (56.5%), 39,519 (39.8%), 36,150 (36.5%), 24,872 (25.1%) and 41,688 (42.0%) unigenes were found in NR, SwissProt, KEGG, COG, and GO databases, respectively. The remaining 39,180 (39.5%) unmapped unigenes were not identified (Table 2). After searching all unigene sequences against four protein databases (NR, SwissProt, KEGG, and COG) using BLASTx (*E*-value < 0.00001), we identified 57,044 coding sequences (CDSs) and predicted proteins. For unigenes with no BLAST hits, we used ESTScan to predict 6834 CDSs and predicted proteins. The distributions of the CDSs and predicted proteins are shown in Figure 3.

**Table 2:** Number of functional annotations for all unigenes in public databases.

Public Protein Database	Number of Unigenes	Percentage (%)
NR	55,996	56.5
Swiss-Prot	39,519	39.8
KEGG	36,150	36.5
COG	24,872	25.1
GO	41,668	42.0
All	59,994	60.5
Unmapped	39,180	39.5

**Figure 4:**
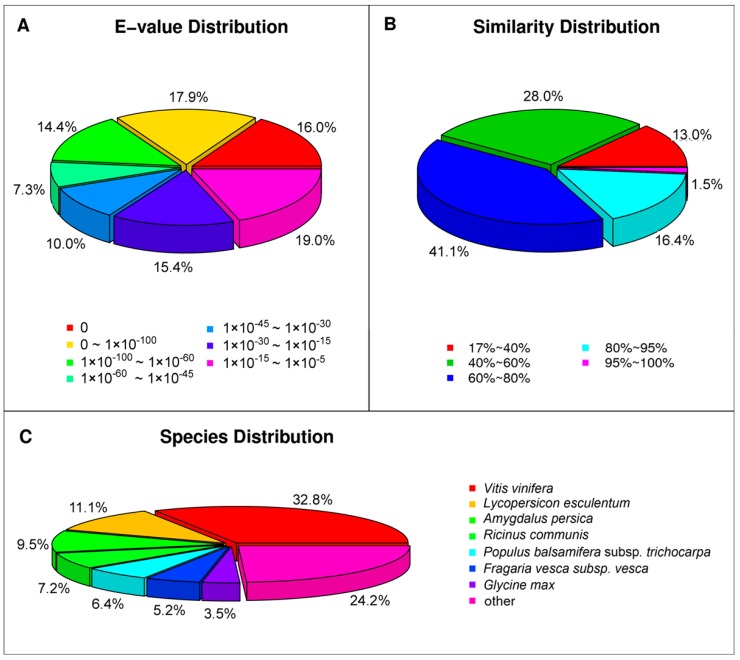
Characteristics of homology search of query sequences aligned by BLASTx to the NCBI nonredundant (NR) database. (**A**) *E*-value distribution of BLAST hits for matched unigene sequences, using an *E*-value cutoff of 1.0 × 10^−^^5^; (**B**) Similarity distribution of top BLAST hits for each unigene; (**C**) Species distribution of the top BLAST hits.

For the NR annotations, 55,996 of the unigenes were found to be matched in the database. For the *E*-value distribution, 44.4% of moderate homolog sequences ranged from 1.0 × 10^−^^5^ to 1.0 × 10^−^^45^, while 55.6% of sequences had a threshold *E*-value less than 1.0 × 10^−^^45^, showing strong homology (Figure 4A). The identity distribution pattern showed that 59% of the sequences had a similarity higher than 60%, while 41% showed similarity between 17% and 60% (Figure 4B). The majority of the annotated sequences corresponded to the known protein sequences of plant species. However, we found that 32.8% of the unigenes had similar sequences to proteins from *Vitis vinifera*, followed by *Lycopersicon esculentum* (11.1%), *Amygdalus persica* (9.5%), *Ricinus communis* (7.2%) and *Populus balsamifera* subsp*. Trichocarpa* (6.4%; Figure 4C). In addition, the other 24.2% unigenes had matches with other plant species, such as *Cucumis sativus* and *Medicago truncatula.* This result suggested that the sequences of the *P. subulata* transcripts generated in the present study were assembled and annotated properly.

**Figure 5:**
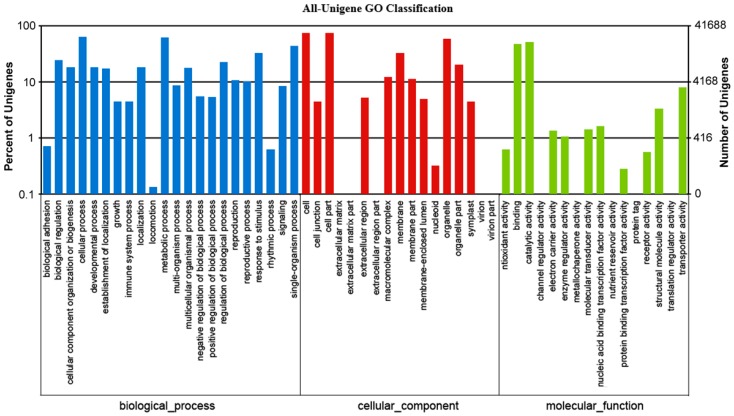
Histogram of gene ontology (GO) classification. The results are summarized in three main categories: Biological processes (**blue**), cellular components (**brown**) and molecular functions (**green**). The *y*-axis on the right side indicates the percent of unigenes in a category, and the *y*-axis on the left side indicates the number of unigenes.

Based on the NR annotation, we used the GO annotation to classify the possible functions of the unigenes. GO annotation is an international classification system that provides controlled vocabulary for descriptions of gene functions [16]. A total of 41,688 (42.0% of all unigenes) unigenes were successfully assigned to at least one GO term. All the GO terms were classified into 56 functional groups, including three main categories: biological processes, cellular components, and molecular functions (Figure 5). Among biological processes, transcript sequences assigned to cellular (26,638), metabolic (25,871) and single-organism processes (18,247) were the most abundant. For cellular components, proteins were mostly assigned to cell (31,028), cell part (31,024) and organelle (24,793) categories. Within the molecular function category, the majority of the GO terms were predominantly assigned to binding (19,903), catalytic activity (21,442) and transporter activity (3303) (Figure 5). Similar results were found in many plant species [11,12,13], suggesting that the DEGs were responsible for fundamental biological metabolism and regulation.

**Figure 6:**
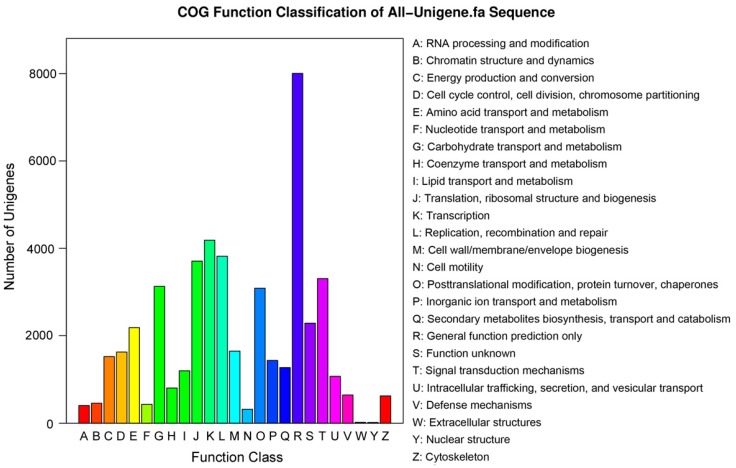
Clusters of orthologous groups (COG) functional classification. A total of 24,872 unigenes were assigned to 25 classifications. The capital letters along the *x*-axis indicate the COG categories as listed on the right of the histogram, and the *y*-axis indicates the number of unigenes.

To further predict gene function and to evaluate the integrality of the *P. Subulata* transcriptome, all unigenes were searched against the COG database. Overall, 24,872 (25.1% of all unigenes) unigenes were assigned COG classifications (Figure 6). By classifying the possible functions of these unigenes, we grouped the unigenes into 25 functional categories. The largest category was “General function prediction only” (8804 of 24,872 unigenes, about 35.4%), followed by “Transcription” (4186 unigenes, 16.8%), “Replication, recombination and repair” (3820, 15.4%), “Translation, ribosomal structure, and biogenesis”, (3708, 14.9%), and “Signal transduction mechanisms” (3307, 13.3%). The categories of “Nuclear structure” (19, 0.07%), “Extracellular structures” (21, 0.08%) and “Cell motility” (320, 1.29%) had the fewest responding unigenes. Additionally, 2287 (9.10%) unigenes were annotated as “Function unknown” (Figure 6).

### 2.3. Analysis of Potential Differentially Expressed Genes (DEGs)

**Figure 7:**
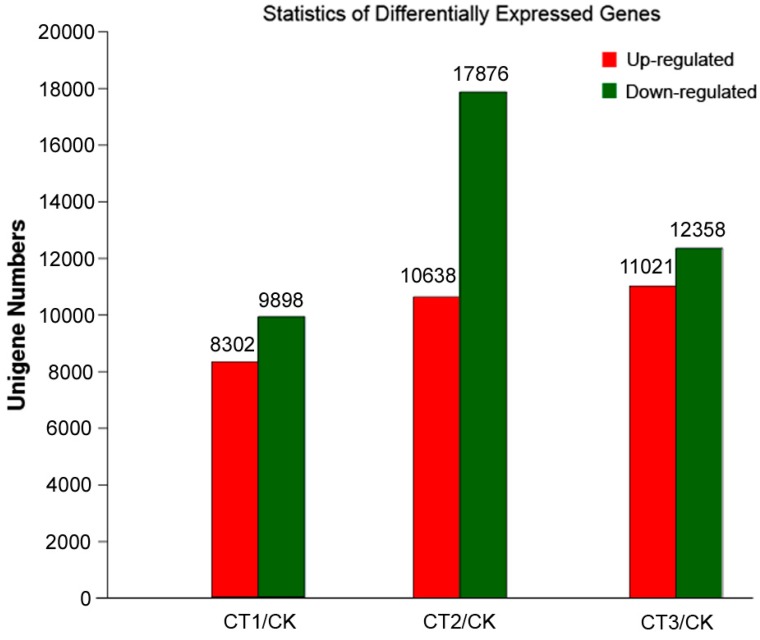
Differentially expressed genes in *P. subulata* under low temperatures. The numbers of up-regulated and down-regulated genes in cold-treated (CT) samples are shown. We used “false discovery rate ≤0.001 and the absolute value of log_2_Ratio ≥1” as the threshold to judge the significance of differences in gene expression. Control (CK) plants were grown at 20 °C. CT1, CT2 and CT3 plants were incubated at 4, 0 and −10 °C, respectively.

**Figure 8:**
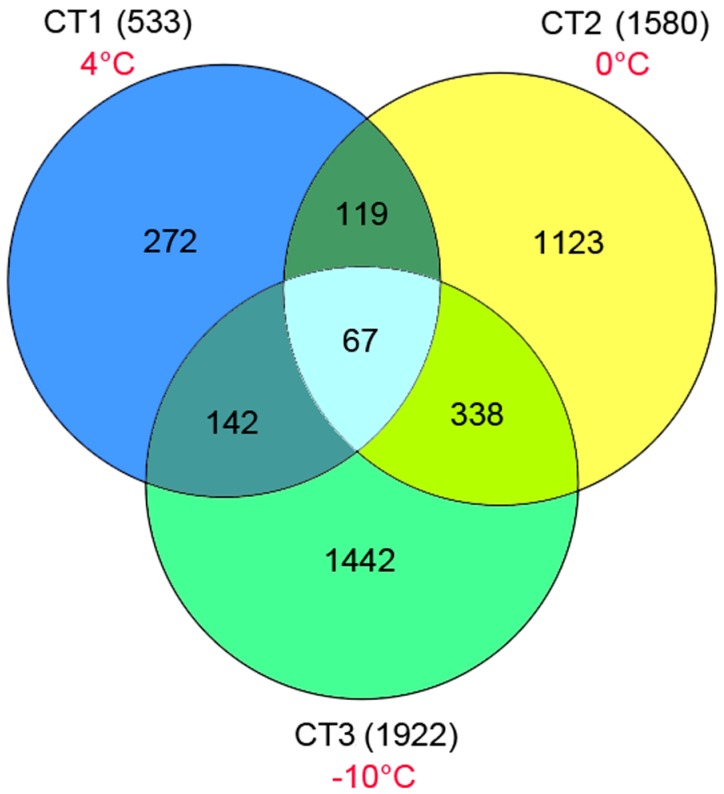
The numbers of up-regulated genes expressed only in the three CT samples (4, 0, and –10 °C).

To identify different expression levels of unigenes, we used the reads per kb per million reads (RPKM) method to calculate the expression levels of the unigenes from CT and CK samples. Screening of DEGs (FDR ≤ 0.001 and ratios larger than 2), the results showed that the numbers of both up- and down-regulated genes changed with reduction of the temperature. The distribution of transcript changes of unigenes is shown in Figure S2. Among all DEGs, 8302 were induced by cold, while 9898 were down-regulated after incubation at 4 °C (CT1; Figure 7 and Table S1). After incubation at 0 °C (CT2), 10,638 genes were up-regulated and 17,876 were down-regulated (Figure 7 and Table S2). After incubation at −10 °C (CT3), 11,021 genes were up-regulated, and 12,358 were down-regulated (Figure 7 and Table S3). We also identified a total of 3417 up-regulated unigenes expressed only in the CT samples (Table S4). Among them, 533, 1580 and 1922 unigenes were found in CT, CT2 and CT3, respectively (Figure 8). Some of these unigenes had no homologs in the NCBI database, suggesting that they may represent new cold-related transcripts that have not been reported in model plants.

### 2.4. Analysis of DEGs Related to Metabolism

To understand the biological function of DEGs, we performed metabolic pathway enrichment analysis using the KEGG database. A total of 21,155 up-regulated unigenes and 22,737 down-regulated unigenes were identified after cold treatment. All DEGs were related to 128 metabolic pathways (Table S5). Among the mapped pathways, 43, 33 and 61 pathways were significantly enriched (*p* ≤ 0.05) after cold treatment. We listed the 10 most significantly enriched pathways (Table 3). After incubation at 4 °C, the four most significantly enriched pathways were plant-pathogen interactions (Figure S3), ether lipid metabolism (Figure S4), biosynthesis of secondary metabolites and glycerophospholipid metabolism pathways (Figure S5). After incubation at 0 °C, the ribosome and plant-pathogen interaction pathways were significantly identified as the top two pathways. After incubation at −10 °C, we found that biosynthesis of secondary metabolites, metabolic pathways, phenylpropanoid biosynthesis, ether lipid metabolism and glycerophospholipid metabolism were significantly enriched (Table 3).

**Table 3:** The 10 most significantly enriched pathways of differentially expressed genes under cold treatment.

Sample	Pathway ID	Pathway	All Genes with Pathway Annotation	DEGs Genes with Pathway Annotation	*p* Value
**CT1/CK**	ko04626	Plant-pathogen interaction	2388 (6.61%)	691 (9.14%)	4.63 × 10^−22^
	ko00565	Ether lipid metabolism	791 (2.19%)	278 (3.68%)	6.63 × 10^−21^
	ko01110	Biosynthesis of secondary metabolites	4516 (12.49%)	1171 (15.49%)	2.76 × 10^−18^
	ko00564	Glycerophospholipid metabolism	1034 (2.86%)	332 (4.39%)	9.88 × 10^−18^
	ko00945	Stilbenoid, diarylheptanoid and gingerol biosynthesis	319 (0.88%)	134 (1.77%)	1.06 × 10^−17^
	ko04144	Endocytosis	1097 (3.03%)	346 (4.58%)	3.95 × 10^−17^
	ko00941	Flavonoid biosynthesis	391 (1.08%)	153 (2.02%)	1.23 × 10^−16^
	ko01100	Metabolic pathways	8740 (24.18%)	2088 (27.62%)	4.49 × 10^−15^
	ko04075	Plant hormone signal transduction	1752 (4.85%)	499 (6.6%)	8.37 × 10^−15^
	ko03050	Proteasome	166 (0.46%)	79 (1.04%)	1.60 × 10^−14^
**CT2/CK**	ko03010	Ribosome	1440 (3.98%)	593 (5.23%)	6.57 × 10^−16^
	ko04626	Plant-pathogen interaction	2388 (6.61%)	881 (7.77%)	1.47 × 10^−9^
	ko00908	Zeatin biosynthesis	384 (1.06%)	176 (1.55%)	1.71 × 10^−9^
	ko00460	Cyanoamino acid metabolism	355 (0.98%)	163 (1.44%)	5.71 × 10^−9^
	ko00945	Stilbenoid, diarylheptanoid and gingerol biosynthesis	319 (0.88%)	145 (1.28%)	8.29 × 10^−8^
	ko00940	Phenylpropanoid biosynthesis	812 (2.25%)	320 (2.82%)	5.48 × 10^−7^
	ko00904	Diterpenoid biosynthesis	188 (0.52%)	91 (0.8%)	7.60 × 10^−7^
	ko00900	Terpenoid backbone biosynthesis	284 (0.79%)	128 (1.13%)	8.04 × 10^−7^
	ko00944	Flavone and flavonol biosynthesis	179 (0.5%)	87 (0.77%)	1.06 × 10^−6^
	ko01110	Biosynthesis of secondary metabolites	4516 (12.49%)	1552 (13.69%)	1.97 × 10^−6^
**CT3/CK**	ko01110	Biosynthesis of secondary metabolites	4516 (12.49%)	1521 (16.37%)	9.61 × 10^−38^
	ko01100	Metabolic pathways	8740 (24.18%)	2704 (29.11%)	3.08 × 10^−37^
	ko00940	Phenylpropanoid biosynthesis	812 (2.25%)	351 (3.78%)	4.43 × 10^−28^
	ko00565	Ether lipid metabolism	791 (2.19%)	325 (3.5%)	9.99 × 10^−22^
	ko00564	Glycerophospholipid metabolism	1034 (2.86%)	403 (4.34%)	1.47 × 10^−21^
	ko00500	Starch and sucrose metabolism	986 (2.73%)	376 (4.05%)	2.23 × 10^−18^
	ko00941	Flavonoid biosynthesis	391 (1.08%)	179 (1.93%)	5.66 × 10^−18^
	ko04144	Endocytosis	1097 (3.03%)	386 (4.16%)	9.57 × 10^−13^
	ko00908	Zeatin biosynthesis	384 (1.06%)	161 (1.73%)	2.54 × 10^−12^
	ko00945	Stilbenoid, diarylheptanoid and gingerol biosynthesis	319 (0.88%)	137 (1.47%)	1.38 × 10^−11^

### 2.5. Identification of Major Genes Involved in Cold Stress

Next, we concentrated on analysis of genes up-regulated in response to cold stress in *P. subulata*. Functional annotations of up-regulated unigenes showed that some unigenes were closely related to cold stress, including cold-inducible transcription factors, cold-induced proteins, antioxidant enzymes, osmoregulation proteins, and proteins related to Ca^2+^ and ABA signaling [12,13,17,18]. We selected 20 cold-related unigenes and estimated their expression levels by the RPKM method (Table 4). Among these genes, dehydration-responsive element binding protein (DREB), ethylene-responsive element binding factor (AP2/ERF), and the transcription factors MYB (v-myb avian myeloblastosis viral oncogene homolog) and ICE1 (inducer of CBF expression 1) were found to be significantly induced by cold stress in *P. subulata.* These transcription factors have been described as major factors involved in plant cold-stress responses [5,19,20]. Small groups of cold-related proteins, including low temperature-regulated (LT), cold-regulated (COR), and shock proteins were involved in responses to cold stimuli. Genes within the LT and COR groups have been previously reported to be involved in the cold stress response process [21,22]. Additionally, some antioxidant enzymes and osmoregulation proteins were identified, including superoxide dismutase (SOD), peroxidase (POD), catalase (CAT), glutathione peroxidase (GPX), water stress-induced protein, and sugar transporter. Under cold stress, reactive oxygen species (ROS) accumulate, and water becomes imbalanced in plants. These phenotypes are harmful to both the membrane and related biochemical reactions [23]. The accumulation of antioxidant enzymes and osmoregulation proteins contributes to the stabilization of membranes as these molecules protect membranes against cold injury [18,24]. Moreover, we also found that ABA-hydroxylase and calcium-dependent protein kinase (CDPK) were up-regulated (Table 4). These genes may be involved in the modification and processing of ABA and Ca^2+^ signaling molecules, which play important roles in signal transduction under cold stress [18,25]. Finally, many unigenes with no known homologs in the NR were also found. These unigenes may represent novel genes specifically expressed in *P. subulata* or may participate in specific regulatory mechanisms associated with cold adaptation in *P. subulata*. However, further studies are needed to verify and characterize these unigenes.

**Table 4:** Analysis of differences in the expression of genes up-regulated in response to cold stress in *Phlox subulata*.

Gene ID	Annotation	Expression Difference Analysis (Log_2_(CT_RPKM/CK_RPKM))		
		CT1/CK	CT2/CK	CT3/CK
Unigene933_All	Dehydration-responsive element-binding protein 1B	0.49	4.23	5.12
Unigene30882_All	Dehydration-responsive element-binding protein 1D	1.49	4.22	5.23
Unigene13555_All	AP2/ERF domain-containing transcription factor	1.56	3.50	4.03
CL11425.Contig1_All	Transcription factor MYB1R1	1.91	0.47	1.49
CL10927.Contig2_All	Transcription factor ICE1	0.56	1.88	2.18
CL12801.Contig1_All	Cold-inducible protein	3.46	7.43	4.37
CL3749.Contig3_All	Cold-inducible protein	5.33	10.29	9.68
CL10279.Contig1_All	Cold acclimation protein COR413PM2	2.55	1.36	1.03
Unigene18542_All	Cold acclimation protein COR413IM2	1.80	1.56	1.41
CL6095.Contig8_All	Cold regulated COR18	2.41	4.13	2.24
Unigene52767_All	Cold shock protein CS66	6.88	9.67	12.23
CL10842.Contig2_All	Cold-inducible RNA-binding protein	3.56	5.61	3.58
CL12984.Contig6_All	Low-temperature-induced 65 kDa protein	1.56	6.35	5.07
CL10225.Contig1_All	Heat shock protein	0.60	1.26	2.75
Unigene52380_All	Superoxide dismutase	4.03	3.65	11.15
Unigene9176_All	Peroxidase 52	1.02	2.48	1.26
Unigene701_All	Peroxidase 57	1.53	2.51	2.75
CL12499.Contig1_All	Catalase isozyme B	1.47	3.21	5.69
CL6576.Contig2_All	Glutathione peroxidase GPX6	1.52	2.68	1.68
CL11774.Contig1_All	Water stress-induced protein ER5	3.46	6.19	4.78
Unigene8303_All	Bidirectional sugar transporter N3	7.75	6.29	9.03
CL7748.Contig2_All	Bidirectional sugar transporter SWEET11	0.66	2.49	2.04
CL1443.Contig6_All	Abscisic acid-hydroxylase	0.80	3.33	2.93
Unigene58_All	Calcium-dependent protein kinase	0.46	2.05	1.26

## 3. Experimental Section

### 3.1. Plant Materials and Cold Treatments

*P. subulata* L. plants were grown in the nursery for three months at the Heilongjiang Academy of the Sciences (China; 128.4°E, 45.0°N). Subsequently, control plants were grown in a growth chamber under a 12/12-h light/dark photoperiod (20–40 μm·s^−1^·m^−2^ light intensity) at 20 °C. For cold treatments, plants were transferred to 4, 0 and −10 °C under the same light source. Roots, stems, and leaves of three CT and CK plants were collected simultaneously after treatment for three days and immediately stored at −80 °C until required for RNA extraction.

### 3.2. RNA Extraction, cDNA Library Construction, and Transcriptome Sequencing

Total RNA from each sample was isolated using TRIzol reagent (Invitrogen, Carlsbad, CA, USA) according to the manufacturer’s instructions. The RNA samples were examined using a NanoDrop ND-8000 Spectrophotometer (NanoDrop, Wilmington, DE, USA); the A260/A280 ratios of four samples ranged from 1.8 to 2.0. The integrity of the RNA samples was assessed with an Agilent 2100 Bioanalyzer (Agilent, Palo Alto, CA, USA). The RNA sample (mixed RNA of root, stem, leaf according to 1:1 proportion) was sent to the Beijing Genomic Institute (BGI, Shenzhen, China) for RNA sequencing. Accordingly, cDNA library construction and Illumina transcriptome sequencing were performed following the methods described by Shu *et al.* [14].

### 3.3. De Novo Assembly and Functional Annotation

Raw reads produced from sequencing machines contained dirty reads with adapters or low-quality bases. These data negatively affect bioinformatics analysis. Therefore, dirty raw reads were discarded. *De novo* assembly of the transcriptome was achieved using the short-reads assembly program, Trinity [15]. Trinity combined reads with a certain length of overlap to form longer fragments, known as contigs. These contigs were subjected to sequence clustering to form longer sequences. Such sequences were defined as unigenes.

All of the assembled unigenes were compared with the publicly protein databases, including NR, Swiss-Prot protein, KEGG, COG and GO databases, using BLASTx analysis with a cut-off *E*-value of 10^−5^. The best alignments were used to identify the sequence direction of the assembled unigenes. Unigenes were first aligned to protein databases in the priority order of NR, Swiss-Prot, KEGG and COG. When unigenes could not be aligned using these databases, ESTScan software was introduced to determine the sequence direction [26]. For the NR annotations, the BLAST2GO program was used to obtain GO annotations of assembled unigenes for describing biological processes, molecular functions, and cellular components [27]. After obtaining GO annotations for assembled unigenes, WEGO software [28] was used to determine GO functional classifications for understanding the distribution of unigene functions. Unigenes were aligned to the COG database to predict and classify possible functions of the unigenes. In the final step, KEGG pathway [29] annotations were performed to analyze the metabolic pathways and functions of unigenes.

### 3.4. Differential Expression Analysis of Unigenes

RPKM [30] was used to calculate unigene expression levels, which eliminated the influence of gene length and sequencing level. The *RPKM* method formula was:

(1) $$RPKM=\frac{10^{6}C}{NL/10^{3}}$$

where *C* is the number of reads that uniquely aligned to one unigene; *N* is the total number of reads that uniquely aligned to all unigenes; and *L* is the base number in the CDS of one unigene.

The FDR control method [31] was used in multiple hypothesis testing to correct for *p* values. After the FDR was obtained, the ratio of RPKMs was used to calculate the fold-change in the expression of unigenes in two samples simultaneously. The smaller the FDR and the larger the ratio, the larger was the difference in the expression level between the two samples. In our analysis, DEGs were screened with an FDR threshold of 0.001 or less and an absolute value of the log_2_ ratio of 1 or more [32]. For pathway enrichment analysis, we looked for significantly enriched metabolic pathways or signal transduction pathways containing DEGs by comparison with the whole genome. Finally, some cold stress-related genes were selected and listed.

## 4. Conclusions

In this study, we first reported the transcriptome data of *P. subulata* under cold stress using Illumina/Solexa. The total length of the reads was ~20 Gb. A total of 99,174 unigenes were assembled. The large number of unigenes and their functional annotations provide valuable resource for genetic and genomic studies in *P. subulata* plants. A large number of DEGs involved in cold stress were identified. Further, we identified a total of 3417 up-regulated unigenes expressed only in the cold-treated *P. subulata* plants. Studies of these up-regulated unigenes involved in cold-related metabolic pathways will facilitate the discovery of other cold-resistance genes.

## Supplementary Materials

Supplementary materials can be found at http://www.mdpi.com/1422-0067/16/05/9732/s1.
