# Supplementary material for: De Novo Transcriptome Sequencing of Low Temperature-Treated Phlox subulata and Analysis of the Genes Involved in Cold Stress
Source: Int J Mol Sci. 2015 Apr 29;16(5):9732–48. doi: 10.3390/ijms16059732 (PMC4463614; doi:10.3390/ijms16059732)
Supplement: Supplementary file 1 [file ijms-16-09732-s001.zip › ijms-81079-Supplementary Information/ijms-81079-Supplementary Information-Figures.pdf]

Supplementary Information

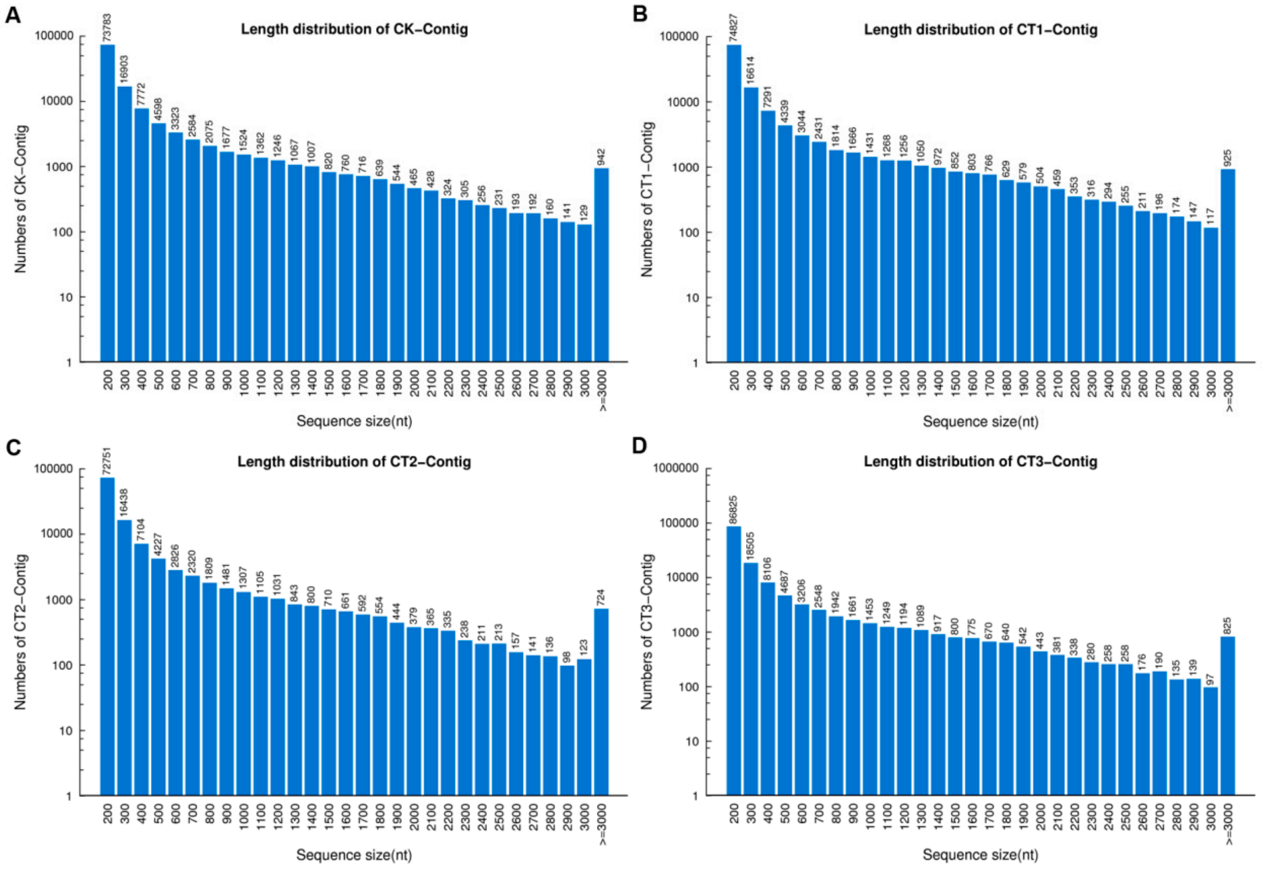

Figure S1. Length distribution of the contigs. The length distribution of contigs of CK sample (**A**); CT1 sample (**B**); CT2 sample (**C**); and CT3 sample (**D**).

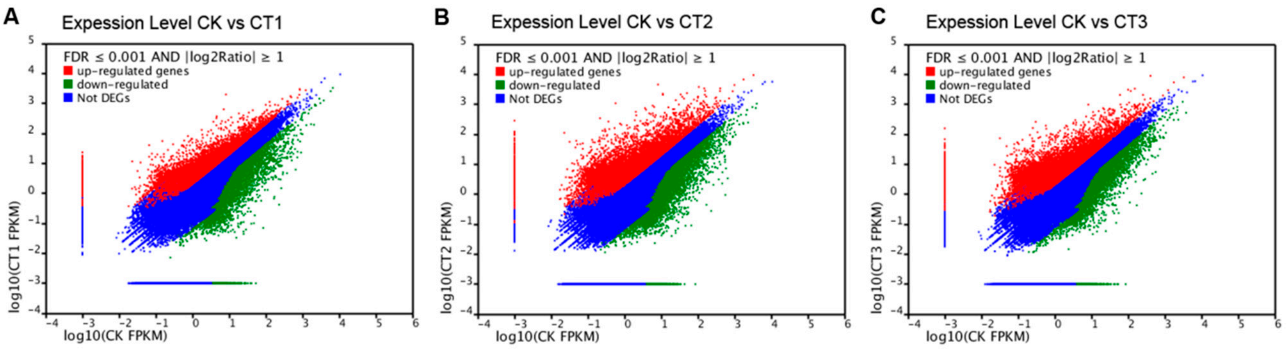

Figure S2. Distribution of differentially expressed genes (DEGs) in cold-treated (CT) samples compared with control sample (CK). We use "FDR (False Discovery Rate) ≤0.001 and the absolute value of log2Ratio ≥1" as the threshold to judge the significance of gene expression difference. Control plants were grown at 20 °C. CT1 (**A**); CT2 (**B**); CT3 (**C**), plants were treated under low temperature for 4, 0 and –10 °C, respectively.

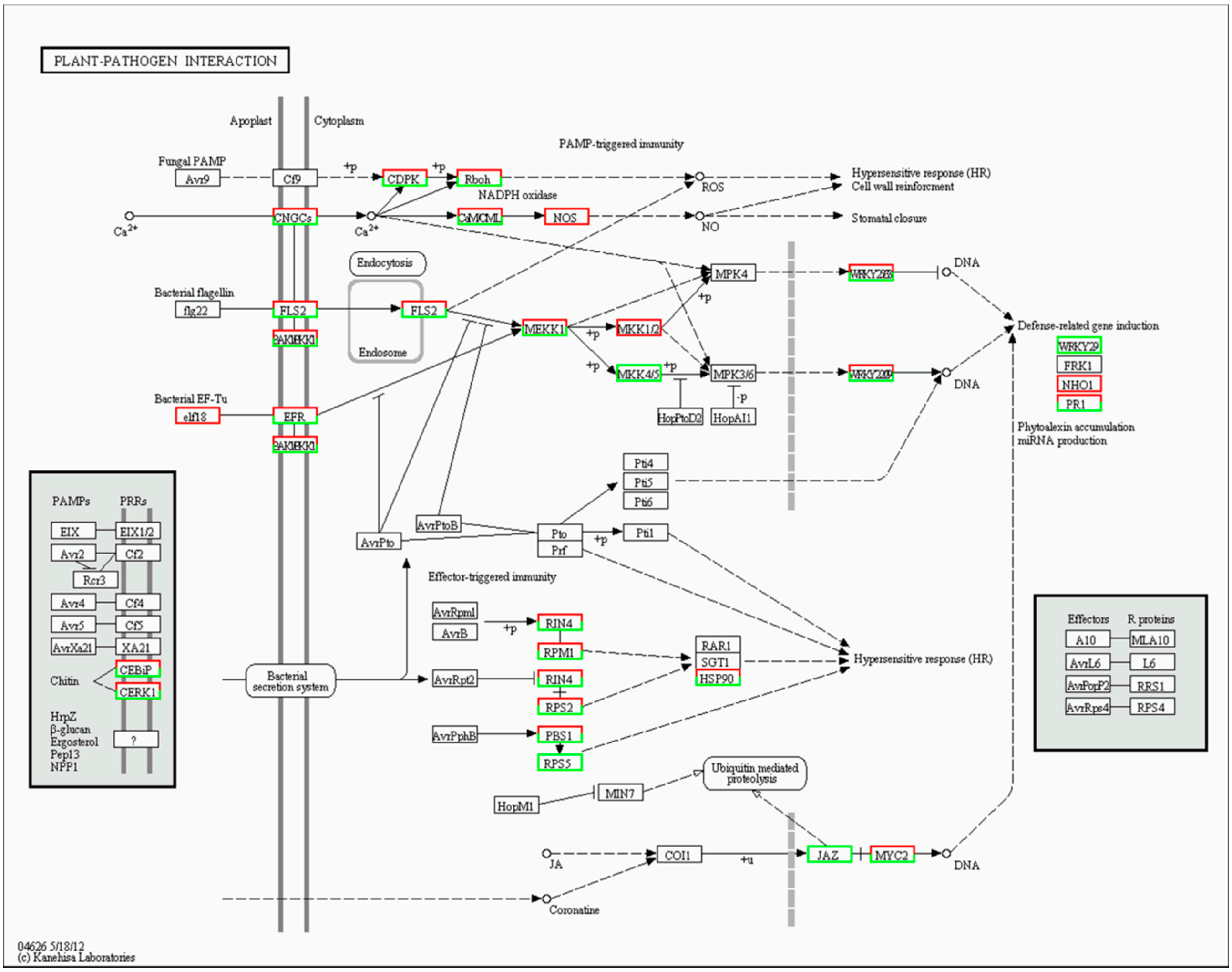

Figure S3. Detailed information of plant-pathogen interaction pathway in KEGG database. In the figure, up-regulated genes are marked with red borders while down-regulated genes are marked with green borders. Non-change genes are marked with black borders.

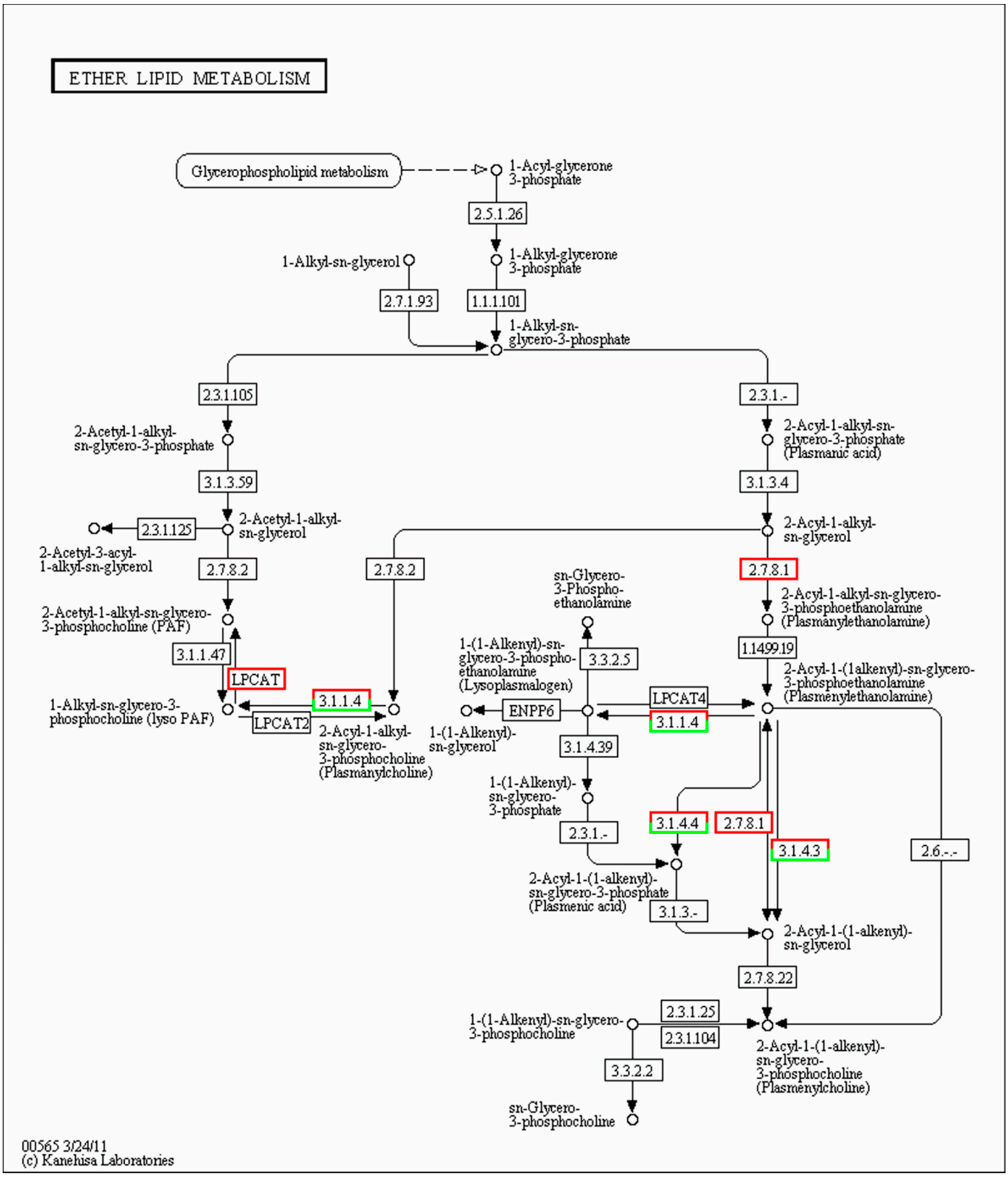

Figure S4. Detailed information of ether lipid metabolism pathway in KEGG database. In the figure, up-regulated genes are marked with red borders while down-regulated genes are marked with green borders. Non-change genes are marked with black borders.

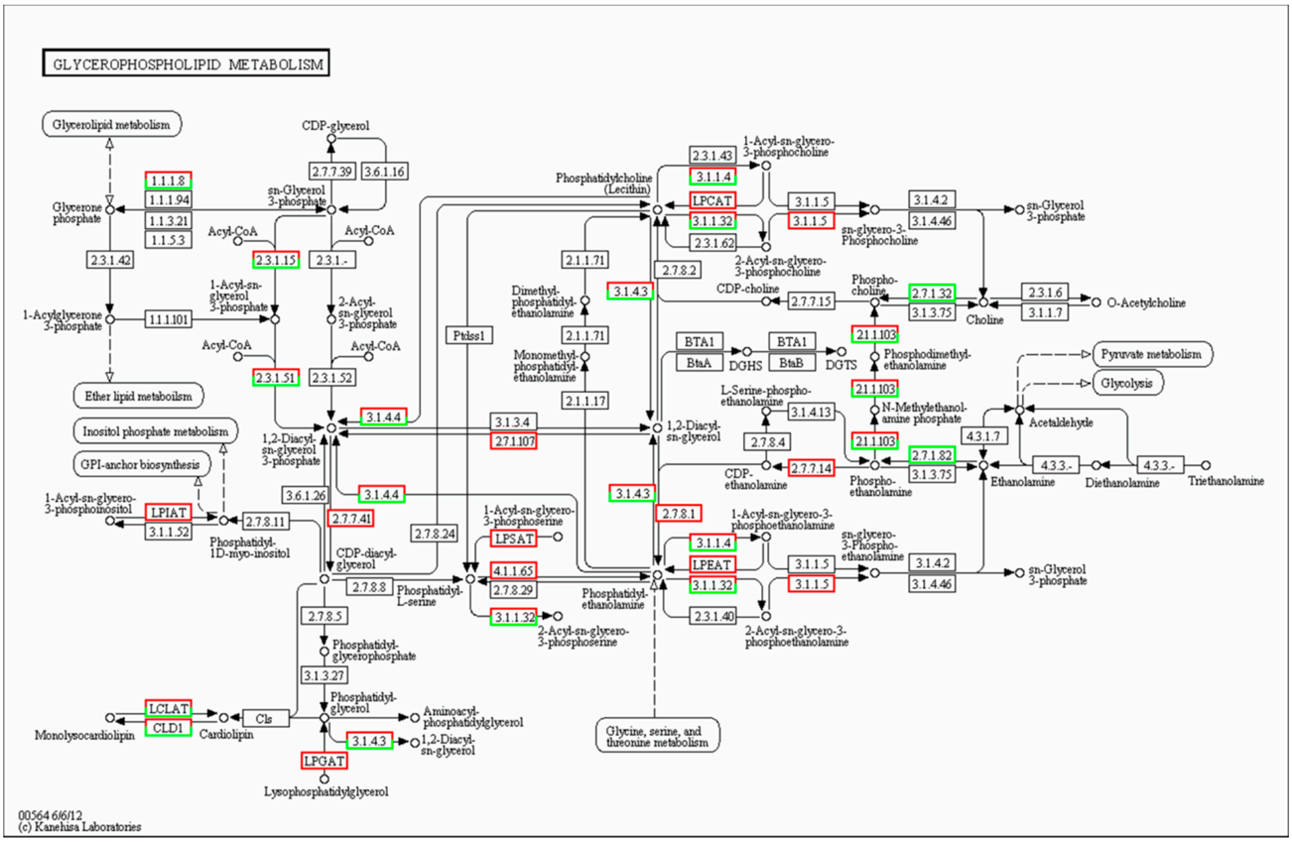

Figure S5. Detailed information of glycerophospholipid metabolism pathway in KEGG database. In the figure, up-regulated genes are marked with red borders while down-regulated genes are marked with green borders. Non-change genes are marked with black borders.
